# A Novel Gd-DTPA-conjugated Poly(L-γ-glutamyl-glutamine)-paclitaxel Polymeric Delivery System for Tumor Theranostics

**DOI:** 10.1038/s41598-017-03633-9

**Published:** 2017-06-19

**Authors:** Lipeng Gao, Jinge Zhou, Jing Yu, Qilong Li, Xueying Liu, Lei Sun, Ting Peng, Jing Wang, Jianzhong Zhu, Jihong Sun, Weiyue Lu, Lei Yu, Zhiqiang Yan, Yiting Wang

**Affiliations:** 1 Institute of Biomedical Engineering and Technology, Shanghai Engineering Research Center of Molecular Therapeutics and New Drug Development, School of Chemistry and Molecular Engineering, East China Normal University, Shanghai, 200062 China; 2 Department of Radiology, Sir Run Run Shaw Hospital, School of Medicine, Zhejiang University, Hangzhou, 310016 China; 3 Department of Pharmaceutics, School of Pharmacy, Fudan University, Ministry of Education, Shanghai, 201203 China; 4 Key Laboratory of Smart Drug Delivery, Fudan University, Ministry of Education, Shanghai, 201203 China

## Abstract

The conventional chemotherapeutics could not be traced *in vivo* and provide timely feedback on the clinical effectiveness of drugs. In this study, poly(L-γ-glutamyl-glutamine)-paclitaxel (PGG-PTX), as a model polymer, was chemically conjugated with Gd-DTPA (Gd-diethylenetriaminepentaacetic acid), a T_1_-contrast agent of MRI, to prepare a Gd-DTPA-conjugated PGG-PTX (PGG-PTX-DTPA-Gd) delivery system used for tumor theranostics. PGG-PTX-DTPA-Gd can be self-assembled to NPs in water with a z-average hydrodynamic diameter about 35.9 nm. The 3 T MRI results confirmed that the relaxivity of PGG-PTX-DTPA-Gd NPs (r_1_ = 18.98 mM^−1^S^−1^) was increased nearly 4.9 times compared with that of free Gd-DTPA (r_1_ = 3.87 mM^−1^S^−1^). The *in vivo* fluorescence imaging results showed that PGG-PTX-DTPA-Gd NPs could be accumulated in the tumor tissue of NCI-H460 lung cancer animal model by EPR effect, which was similar to PGG-PTX NPs. The MRI results showed that compared with free Gd-DTPA, PGG-PTX-DTPA-Gd NPs showed significantly enhanced and prolonged signal intensity in tumor tissue, which should be attributed to the increased relaxivity and tumor accumulation. PGG-PTX-DTPA-Gd NPs also showed effective antitumor effect *in vivo*. These results indicated that PGG-PTX-DTPA-Gd NPs are an effective delivery system for tumor theranostics, and should have a potential value in personalized treatment of tumor.

## Introduction

Cancer is a major public health problem in most countries and is the second leading cause of death in the world^1–3^. At present, chemotherapy is still one of the most effective ways to treat cancer in clinic^4^. However, because conventional chemotherapeutic drugs cannot be traced in the body in real time, their distribution in the tumor tissue cannot be known, therefore, they cannot provide timely feedback for the clinical effectiveness of the drug. This is also the main reason why personalized treatment of cancer cannot be achieved. Many scientists believe that this is the major problem in the current clinical chemotherapy of tumor, which needs to be urgently addressed by an effective technology.

Theranostics, which integrate cancer diagnosis and cancer treatment functions, providing an effective and convenient technique for the personalized treatment of cancer^5–7^. Therapeutic strategies such as chemotherapy, nucleic acid delivery, hyperthermia (photothermal ablation), photodynamic, and radiation therapy are combined with one or more imaging functionalities for both *in vitro* and *in vivo* studies^8^. Nanotechnology has provided a new multifunctional platform for the early diagnosis and accurate treatment of cancer and other serious diseases. Nanoparticles (NPs) have been widely used as a carrier for tumor diagnosis and treatment. NPs are generally categorized as either organic or inorganic materials. Especially, the organic NPs have attracted great attention from the scientific community in the past decades, on account of their easy functionalization, biodegradability, and other unique physiochemical properties. For example, Vaidya *et al*. prepared poly(L-glutamic acid) (PGA)-(Gd-DO3A)-mesochlorin e_6_ (Mce_6_) NPs and evaluated the efficacy for cancer MR imaging and cancer treatment^9^. Pan *et al*. developed nanobialys (biotinylated-Mn(III)-labeled nano-bialys) as theranostic agent, which offers the site-specific MR T_1_-weighted molecular imaging with manganese as well as local delivery of potent chemotherapy agents^10^. Although these attempts have achieved tumor diagnosis and treatment, the low relaxivity of magnetic resonance imaging (MRI) contrast agents and short circulating time *in vivo* may cause the limited resolution of contrast enhancement imaging and decreased tumor accumulation. Thus, researchers have been trying to find theranostics strategies with higher relaxivity and longer circulating time to be used for the tumor diagnosis and treatment.

At present, several types of imaging technologies, including computed tomography (CT)^11^, positron emission tomography (PET)^12^, MRI^13, 14^, ultrasonic imaging^15^ and photoacoustic imaging (PA)^16^, have been applied for guiding cancer treatment. Compared with optical and radionuclide imaging, MRI, a non-ionizing radiation-free technique, has been widely used in cancer detection, staging and monitoring because of its high resolution and wide range of functional imaging capabilities^17, 18^. In order to improve the specificity and sensitivity of MRI, contrast agents are generally used to increase the signal intensity. Many different metal contrast agents based on gadolinium (Gd) (Magnevist, ProHance), Fe (Feridex, Endorem) and Mn (Teslascan) are currently available^19–21^. Among them, Magnevist (Gd-DTPA), one of the most commonly used T_1_ contrast agent for clinical MRI^22^. However, free Gd-DTPA cannot be used in tumor imaging due to the lack of enhanced permeability and retention (EPR) effect in tumor tissue.

Paclitaxel (PTX) is a chemotherapeutic drug that has an excellent therapeutic effects for a wide range of cancers, especially for lung cancer, breast cancer and ovarian cancer, etc^23, 24^. But its clinical application has been seriously limited by its low water solubility and the lack of selectivity to tumor tissue. To solve these problems, many new generation of PTX have been developed, including nanoparticles (such as Abraxane), micelles (such as PEG-PLA/PTX micelles), and polymer conjugated drugs. Of these, polymer conjugated drugs can significantly increase the water solubility of PTX, for example, PEG-PTX and Xyotax™ (poly(L-glutamic acid)-paclitaxel (PGA-PTX))^25^, the latter of which have entered the clinical trials. On the basis of PGA-PTX, a polymer conjugated drug poly(L-γ-glutamyl-glutamine)-paclitaxel (PGG-PTX) (PTX, 34.9 wt%)^26–28^ had been developed in our laboratory, which further increased the water solubility of PTX, improved the pharmacokinetic behavior, increased the tolerable dose, reduced the side effects, and improved the anti-tumor effects^29^.

**Figure 1:**
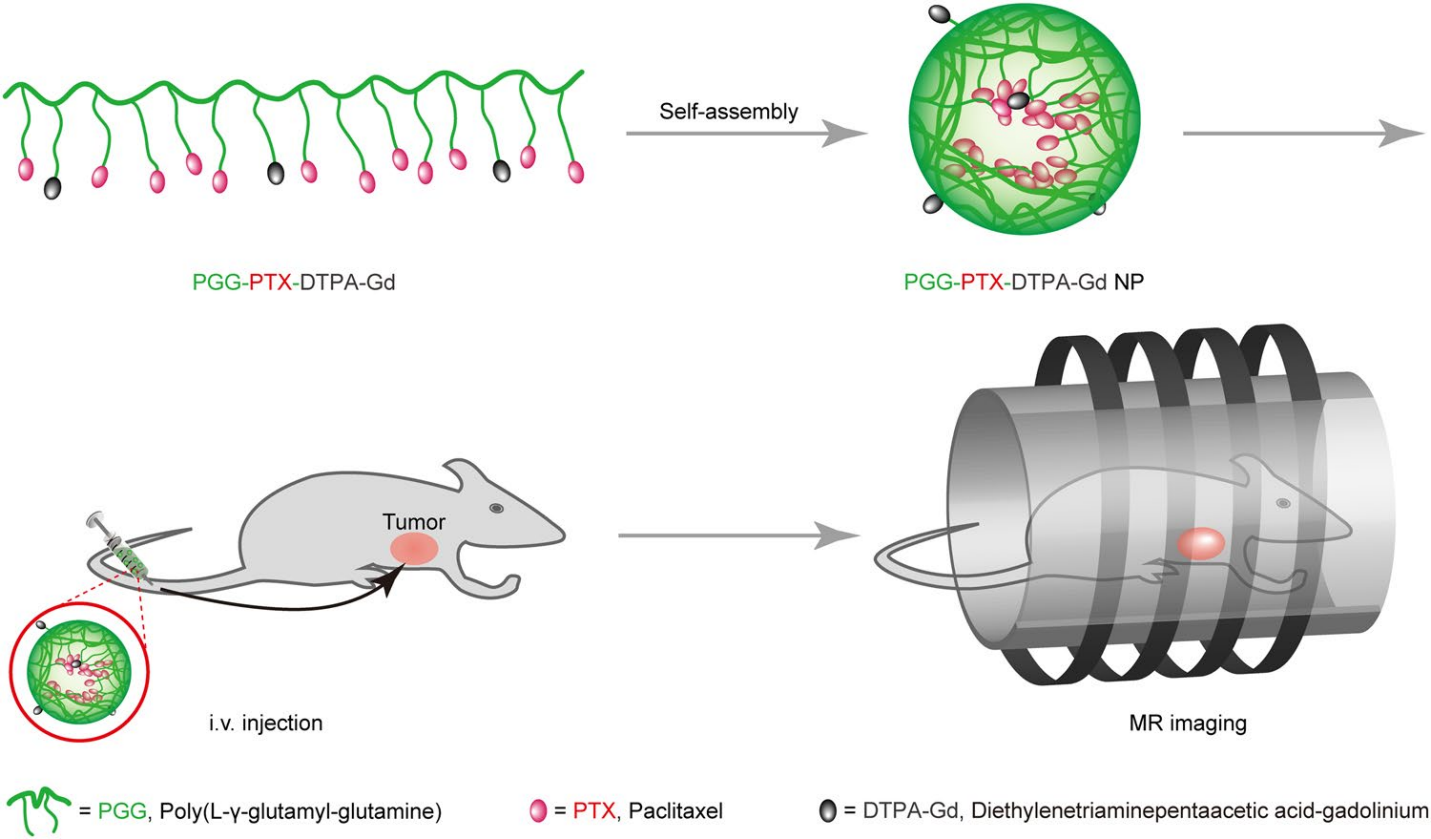
Schematics of the MR imaging of the PGG-PTX-DTPA-Gd.

Based on the above considerations, PGG-PTX, as a model polymer, was chemically conjugated with free Gd-DTPA to prepare a Gd-DTPA conjugated PGG-PTX (PGG-PTX-DTPA-Gd) drug delivery system (DDS) integrated with diagnostic and therapeutic functions for tumor (Fig. 1). The DDS was designed to solve the problem that conventional chemotherapeutic drugs cannot be traced in the body in real time. More importantly, because the prepared polymer can self-assemble into nanoparticles, it can be targeted to tumor tissue by the EPR effect, thereby enhancing the MRI diagnostic response and anti-tumor effect. We characterized the prepared PGG-PTX-DTPA-Gd NPs by ^1^H-NMR, DLS, TEM, and ICP-OES and further evaluated their function of tumor diagnosis and treatment on human NCI-H460 cancer cells *in vitro* and lung cancer animal model *in vivo*.

## Results

### Characterization of PGG-PTX-DTPA-Gd NPs

PGG-PTX-DTPA-Gd were prepared by the complexation of PGG-PTX-DTPA with GdCl_3_. An excess of GdCl_3_, based on DTPA content in the conjugate, was used to ensure complete complexation of DTPA in the conjugate. When GdCl_3_ was added, there was precipitate formed, which was possibly due to the complexation of carboxylic groups of PGG with Gd^3+^ ions. The EDTA disodium salt was used to remove the excess Gd^3+^ ions from the PGG, resulting in a clear reaction mixture at pH 5.0–5.5, adjusted with dilute NaOH. EDTA is a chelating agent, which can form EDTA-Gd complexes with intermediate stability. It can readily strip off the Gd^3+^ ions stuck to PGG and would not affect the formation of DTPA-Gd complexes with higher stability^30^.

### Characterization of ^1^H-NMR and ICP-OES

The chemical structure of the PGG-PTX-DTPA-Gd conjugate is shown in Figure S2A. The green part is PGG, the red is PTX, and the black is Gd-DTPA. The ^1^H-NMR spectra of PGG-PTX (I) and PGG-PTX-DTPA-Gd (II) are shown in Figure S2B. The observation was consistent with ^1^H-NMR studies of the other polymer in D_2_O reported^26^. The ^1^H-NMR spectrum of PGG-PTX-DTPA-Gd showed characteristic peaks of *p*-NH_2_-Bn-DTPA at 7.07 (d, *J* = 7.8 Hz, 2 H) and 6.81 (d, *J* = 7.8 Hz, 2 H) ppm (Figure S2B, a and b, the red arrows), whereas that of PGG-PTX did not show. The ^1^H-NMR results showed that *p*-NH_2_-Bn-DTPA and PGG-PTX had been successfully linked together. The content of the conjugated DTPA was approximately 8%-molar as calculated from the ^1^H-NMR spectrum of PGG-PTX-DTPA-Gd. The Gd content was 0.21 mmol-Gd per gram of polymer conjugate as determined by ICP-OES, which was the same as the content of DTPA estimated from the ^1^H-NMR.

### Characterization of DLS and TEM

Further DLS and TEM studies confirmed the formation of PGG-PTX NPs and PGG-PTX-DTPA-Gd NPs. The particle size distribution of PGG-PTX NPs and PGG-PTX-DTPA-Gd NPs were examined by DLS, and the results are shown in Figure S2C,E. The Z-mean diameter of PGG-PTX NPs and PGG-PTX-DTPA-Gd NPs in water were approximately 29.2 nm and 35.9 nm, and the PDI were 0.177 and 0.183, respectively. Furthermore, the TEM images of PGG-PTX NPs and PGG-PTX-DTPA-Gd NPs exhibited a spherical shape with uniform particle size, as shown in Figure S2D,F. The particle size of PGG-PTX NPs and PGG-PTX-DTPA-Gd NPs observed by TEM were about 23 nm and 28 nm, which were smaller than that determined by DLS, respectively. We speculated that the particle size determined by DLS represents their hydrodynamic diameter, whereas that obtained by TEM represents the collapsed micelles after water evaporation. This result is also consistent with previous report^31^.

### Characterization of Mw

The results are shown in Table S1. The Mw of PGG-PTX and PGG-PTX-DTPA-Gd were about 79.17 (kDa) and 93.89 (kDa), and the PDI were 1.25 and 1.38, respectively.

### Characterization of r_1_ relaxivity

**Figure 2:**
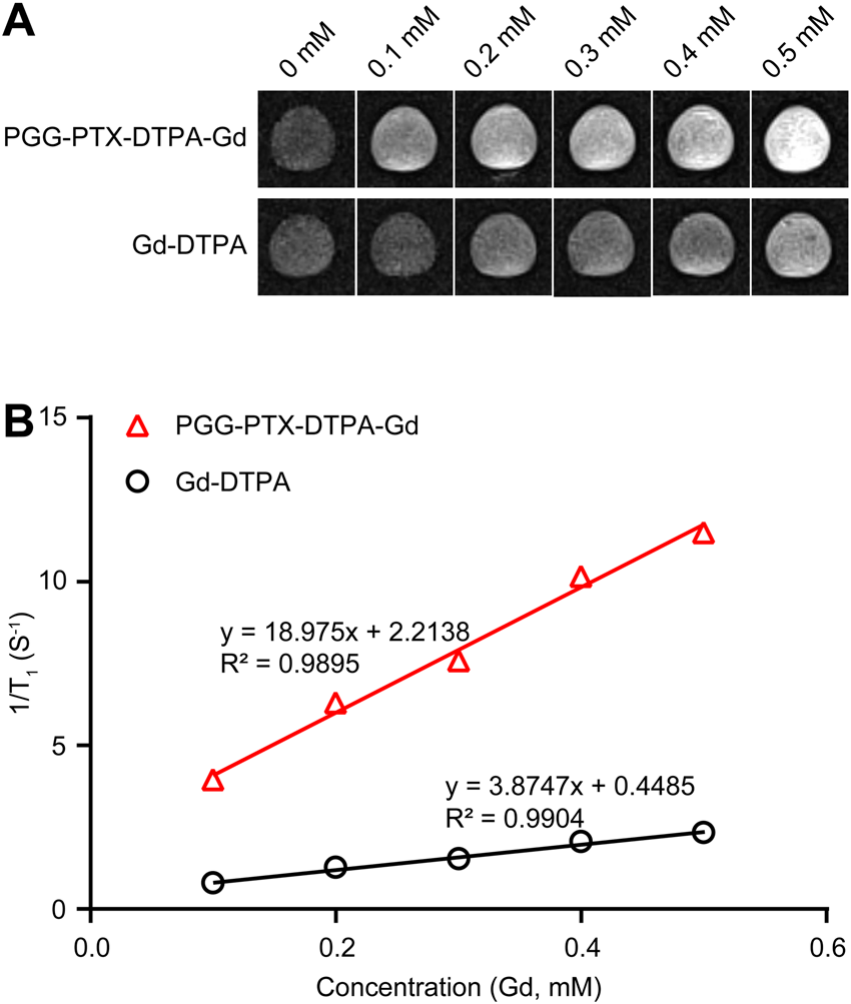
MR enhancement effects of PGG-PTX-DTPA-Gd NPs and free Gd-DTPA. (**A**) T_1_-weighted MR images of PGG-PTX-DTPA-Gd NPs and free Gd-DTPA solutions at 3 T MRI. (**B**) T_1_ relaxivities coefficient of PGG-PTX-DTPA-Gd NPs and free Gd-DTPA characterized by 3 T MRI. PGG-PTX-DTPA-Gd NPs has higher contrast ability than free Gd-DTPA, as the images of PGG-PTX-DTPA-Gd NPs were much brighter than free Gd-DTPA at the same concentration.

The T_1_-weighted MR images of PGG-PTX-DTPA-Gd NPs and free Gd-DTPA solutions at 3 T MRI were shown in Fig. 2. The results showed that PGG-PTX-DTPA-Gd NPs has higher contrast ability than free Gd-DTPA, as the images of PGG-PTX-DTPA-Gd NPs were much brighter than free Gd-DTPA at the same concentration (Fig. 2A). Meanwhile, 3 T MRI results confirmed that the r_1_ value of PGG-PTX-DTPA-Gd NPs (18.98 mM^−1^S^−1^) increased nearly 4.9 times compared with that of free Gd-DTPA (3.87 mM^−1^S^−1^) (Fig. 2B). In addition, the high relaxivity of PGG-PTX-DTPA-Gd NPs may be attributed to a prolongation of the rotational correlation time, due to restricted local motion similar to that of Gd-DTPA bound to macromolecules^32, 33^.

### *In vitro* cellular uptake

**Figure 3:**
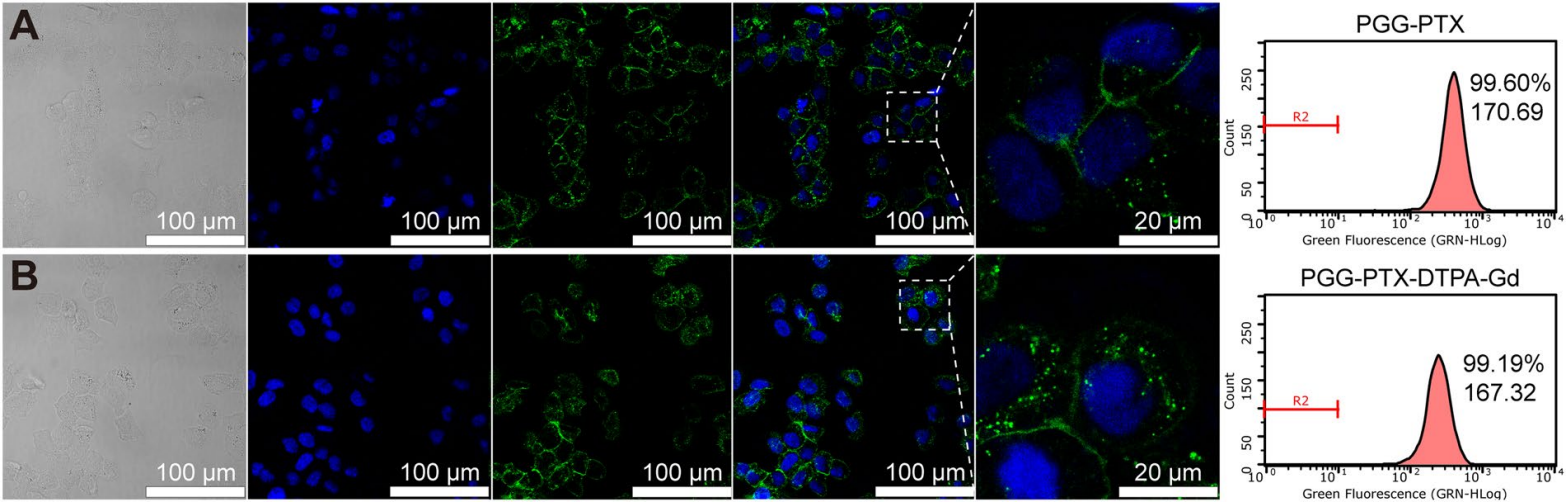
The CLSM images of cellular uptake and flow cytometry for PGG-PTX/DiO NPs (**A**) and PGG-PTX-DTPA-Gd/DiO NPs (**B**). The numbers in flow cytometry pictures represent percentages of DiO-positive cells and mean of fluorescence intensity, respectively. The cellular uptake of PGG-PTX-DTPA-Gd/DiO NPs by NCI-H460 cells was not significantly changed with PGG-PTX/DiO NPs.

To determine the interaction of PGG-PTX-DTPA-Gd NPs with the tumor cells, we observed the cellular uptake of NPs by NCI-H460 cell line using CLSM and flow cytometer. As shown in Fig. 3, the percentages of fluorescent cells in PGG-PTX/DiO NPs and PGG-PTX-DTPA-Gd/DiO NPs were 99.60% and 99.19%, and the mean fluorescent intensities for them were 170.69 and 167.32, respectively. These data indicated that the cellular uptake of PGG-PTX-DTPA-Gd/DiO NPs by NCI-H460 cells did not significantly change compared with that of PGG-PTX/DiO NPs, suggesting that the conjugation of Gd-DTPA to PGG-PTX had no significant influence on the cell uptake of PGG-PTX NPs.

### *In vitro* PTX release

The PTX content of PGG-PTX-DTPA-Gd was determined to be 28.8 wt% by an Agilent 1100 series HPLC system (Agilent Technologies, Santa Clara, CA, USA). Compared to PGG-PTX (PTX, 34.9 wt%), the PTX content of PGG-PTX-DTPA-Gd was decreased, which may be due to the introduction of DTPA-Gd and also the degradation of PGG-PTX during the modification process.

A four-day drug release experiment was performed to compare the PTX release profile of PGG-PTX NPs and PGG-PTX-DTPA-Gd NPs. As shown in Figure S3, PGG-PTX NPs and PGG-PTX-DTPA-Gd NPs showed similar profile of PRX release at all time points. The cumulative release rate of PTX from PGG-PTX-DTPA-Gd NPs reached about 22.5% at 96 h.

### *In vitro* cytotoxicity assays

The cell viability of NCI-H460 cells was evaluated following incubation with PGG-PTX NPs and PGG-PTX-DTPA-Gd NPs. As shown in Figure S4, the results showed that the cell viability of PGG-PTX NPs and PGG-PTX-DTPA-Gd NPs decreased gradually as the drug concentration increased. Moreover, the cells showed similar survival rate when the two drug concentrations are the same. The PGG-PTX-DTPA-Gd NPs showed almost similar cytotoxicity to PGG-PTX NPs, suggesting that the conjugation of Gd-DTPA to PGG-PTX did not significantly affect the cytotoxicity of PGG-PTX NPs.

### *In vivo* fluorescence imaging

**Figure 4:**
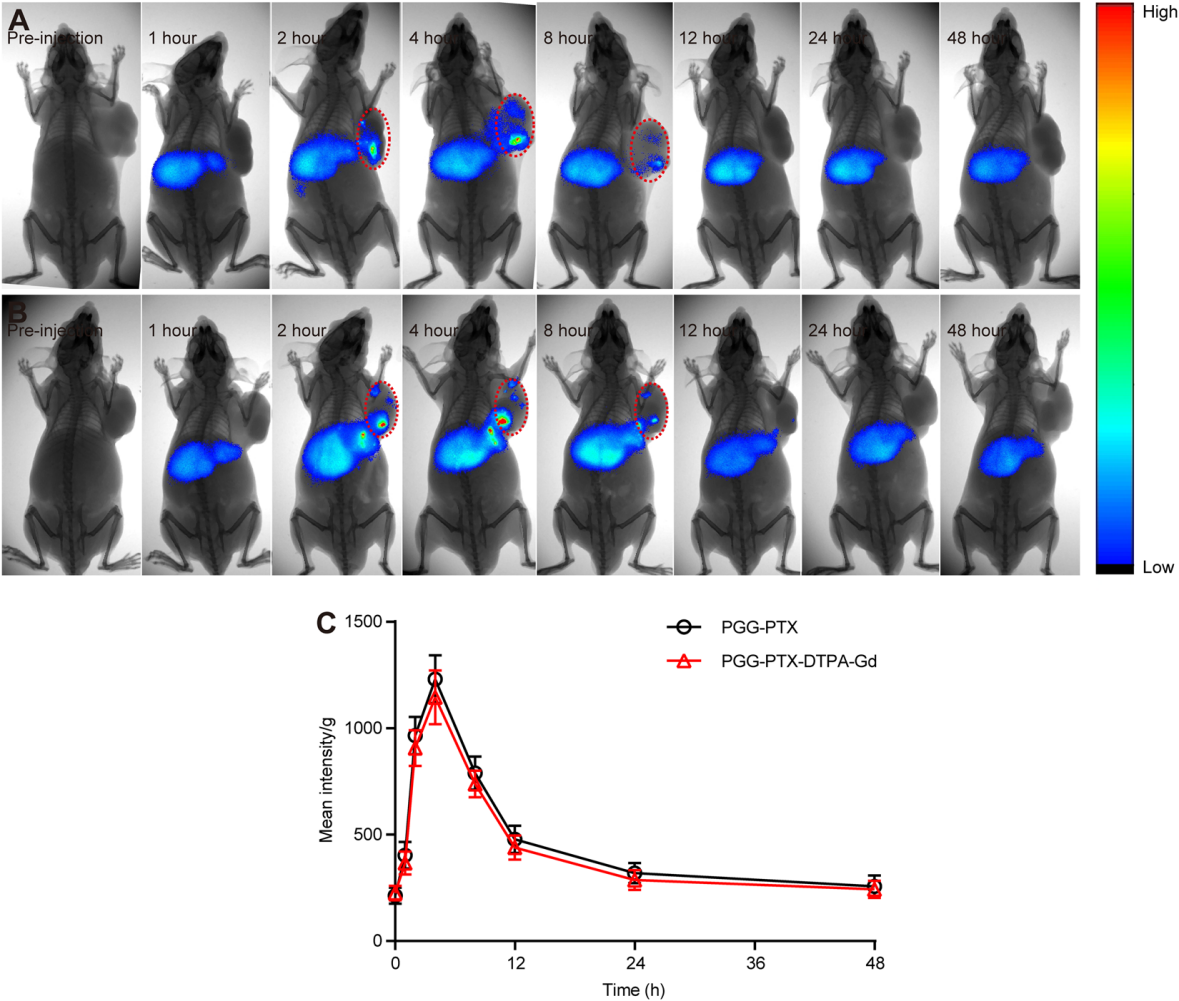
*In vivo* fluorescent imaging of NCI-H460-bearing nude mice (n = 3) after the intravenous injection of PGG-PTX/DiR NPs (**A**) and PGG-PTX-DTPA-Gd/DiR NPs (**B**). Representative *in vivo* fluorescent images of NCI-H460-bearing nude mice following i.v. administration of PGG-PTX-DTPA-Gd/DiR NPs at different time points (pre-injection, 1, 2, 4, 8, 12, 24, 48 hour). Color bar on the right side indicates the signal efficiency of the fluorescence emission. (**C**) Pharmacokinetic profile of DiR in the tumor tissue of animal model after i.v. injection of PGG-PTX-DTPA-Gd/DiR NPs based on the semi-quantitative ROI analysis of *in vivo* fluorescent images. The conjugation of Gd-DTPA to PGG-PTX did not affect the distribution of PGG-PTX NPs in nude mice.

The high *in vitro* MRI contrast performance of PGG-PTX-DTPA-Gd inspired us to pursue their applicability for *in vivo* trials. As shown in Fig. 4, the results showed that there was almost no significant difference between the distribution of PGG-PTX/DiR NPs and PGG-PTX-DTPA-Gd/DiR NPs in tumor-bearing nude mice at different time points. The PGG-PTX-DTPA-Gd/DiR NPs began to accumulate in the tumor tissue 2 h post-injection. The concentration reached the maximum at 4 h post-injection, then gradually decreased and disappeared after 12 h. The pharmacokinetic profile of DiR in the tumor tissue was drawn based on the semi-quantitative ROI analysis of the *in vivo* fluorescent signal per gram of tumor tissue (Fig. 4C). The area under curve (AUC_0–48h_) of PGG-PTX/DiR NPs and PGG-PTX-DTPA-Gd/DiR NPs in the tumor tissue were almost equal, suggesting that the conjugation of Gd-DTPA to PGG-PTX had no significant influence on tumor accumulation of PGG-PTX NPs. In addition, we found that the two NPs tended to be distributed in the liver, which should be a common feature of nano DDSs caused by the phagocytosis by the macrophages in the liver^34^.

### *In vivo* MR imaging

**Figure 5:**
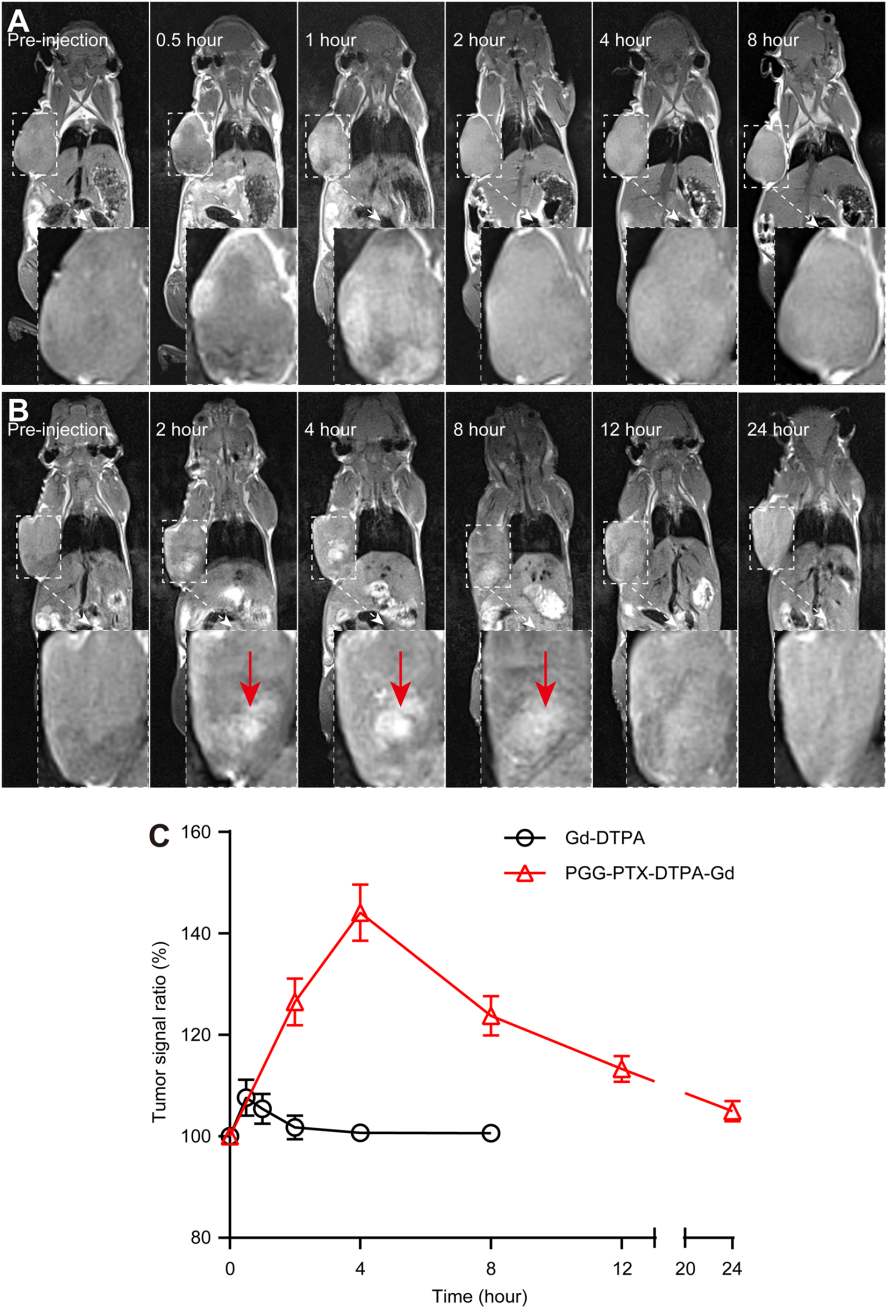
MR imaging of NCI-H460-bearing nude mice (n = 3) with PGG-PTX-DTPA-Gd NPs *in vivo*. Representative *in vivo* T_1_-weighted MR images of NCI-H460-bearing nude mice following i.v. administration of free Gd-DTPA (**A**) at different time points (pre-injection, 0.5, 1, 2, 4, 8 hour) and PGG-PTX-DTPA-Gd NPs (**B**) at different time points (pre-injection, 2, 4, 8, 12, 24 hour). (**C**) Quantitative analysis of T_1_-weighted MR images tumor contrast enhancement after i.v. injection of free Gd-DTPA and PGG-PTX-DTPA-Gd NPs. The average MR relative signal enhancement was measured for each tumor. The higher contrast of tumor positions by PGG-PTX-DTPA-Gd NPs are resulted from the promoted tumor accumulation and the increased relaxivity of PGG-PTX-DTPA-Gd NPs.

In order to explore the tumor diagnostic application of PGG-PTX-DTPA-Gd NPs, we conducted the whole-body animal imaging by intravenously injection into tumor-bearing mice. As shown in Fig. 5, the results showed that in the Gd-DTPA group, the contrast of the whole-body was immediately enhanced, reaching 8% increase in the signal ratio of the tumor to the muscle at 0.5 h post-injection. After that the contrast gradually decreased and basically disappeared after 2 h post-injection. This should be attributed to the rapid renal clearance and the lack of tumor targeting ability of Gd-DTPA due to its low molecular weight. In contrast, after intravenous injection of PGG-PTX-DTPA-Gd NPs, the contrast of the tumor tissue was gradually enhanced, reaching 44% increase in the signal ratio of the tumor to the muscle at 4 h post-injection (Fig. 5C). After that the contrast gradually decreased and basically disappeared after 12 h post-injection. The higher contrast of tumor positions of PGG-PTX-DTPA-Gd NPs might result from the increased tumor accumulation due to the EPR effect (as shown in Fig. 4) and the increased relaxivity of PGG-PTX-DTPA-Gd NPs (as shown in Fig. 2).

### *In vivo* antitumor effect

The *in vivo* anti-tumor efficacy of PGG-PTX-DTPA-Gd NPs was investigated on the NCI-H460 tumor-bearing mice. As shown in Figure S5A, the mean tumor volume of mice in the PGG-PTX NPs and PGG-PTX-DTPA-Gd NPs group reached 1737 mm^3^ and 1641 mm^3^, respectively, which were significantly lower than that in Saline group with 2972 mm^3^ (P < 0.001). The results indicated that both PGG-PTX NPs and PGG-PTX-DTPA-Gd NPs had effective antitumor effect *in vivo*. In addition, the two groups showed similar antitumor effect, suggesting that the conjugation of Gd-DTPA to PGG-PTX had no significant effluence on the antitumor effect of PGG-PTX NPs. Besides, there was a more increase of the mean body weight in the PGG-PTX NPs group (14.3%) and PGG-PTX-DTPA-Gd NPs group (15.5%) than the Saline group (8.9%) (Figure S5B), which is probably resulted from the good physical condition of mice in the two NPs groups due to the tumor suppression.

### Histology verification

The antitumor effect of PGG-PTX-DTPA-Gd NPs *in vivo* was further evaluated by the histological tumor tissue images through H&E staining and TUNEL assay.

### H&E staining

**Figure 6:**
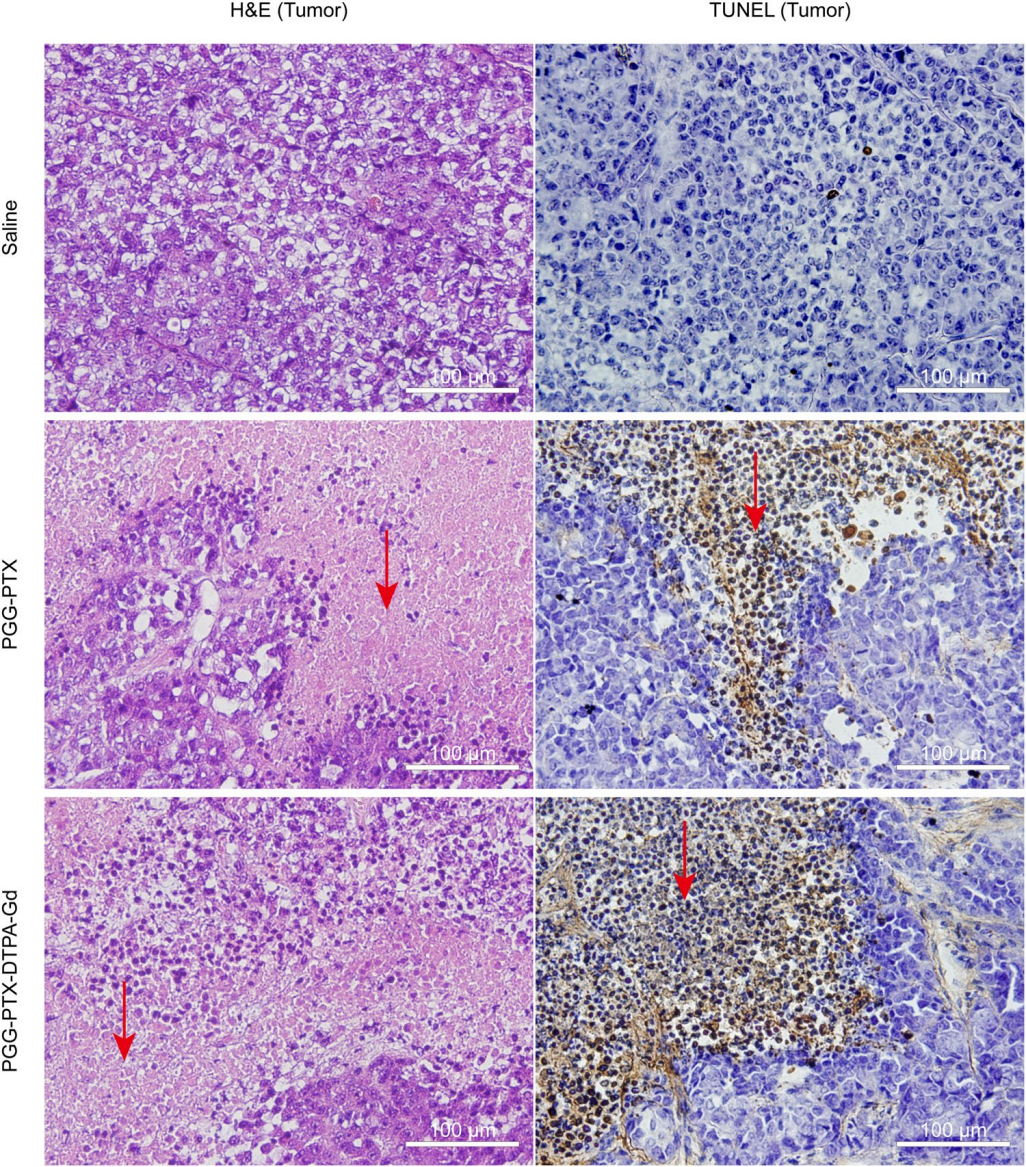
The H&E staining and TUNEL assay for tumor tissues harvested from the mice after the treatment (40x) with Saline (control), PGG-PTX NPs and PGG-PTX-DTPA-Gd NPs. The tumor tissue slides of the PGG-PTX-DTPA-Gd NPs and PGG-PTX NPs group showed a large amount of cell death (H&E staining) and significant apoptosis (TUNEL assay) compared with the Saline group (the red arrow).

The H&E staining assay was performed to evaluate the antitumor effect of PGG-PTX-DTPA-Gd NPs and PGG-PTX NPs from the tumor tissue slides and the histocompatibility from the main organs slides. The tumor tissue slides of the PGG-PTX-DTPA-Gd NPs and PGG-PTX NPs group showed a large amount of cell death compared with the Saline group (Fig. 6, the red arrow). In contrast, the main organs (including heart, liver, spleen, lung, and kidney) of the two NPs groups showed no obvious pathological abnormity compared with those of Saline groups (Figure S6), indicating a good histocompatibility of PGG-PTX-DTPA-Gd NPs. The above H&E staining results are consistent with the *in vivo* antitumor effect.

### TUNEL assay

The tumor tissues of the PGG-PTX-DTPA-Gd NPs groups and the PGG-PTX NPs groups had obvious a large numbers of TUNEL-positive cells, with significant apoptosis compared with the Saline group (Fig. 6, the red arrow). There was no significant difference between PGG-PTX-DTPA-Gd NPs group and PGG-PTX NPs group. The above TUNEL assay results are basically consistent with the above results of *in vivo* antitumor effect (Figure S5).

### Tumor accumulation

**Figure 7:**
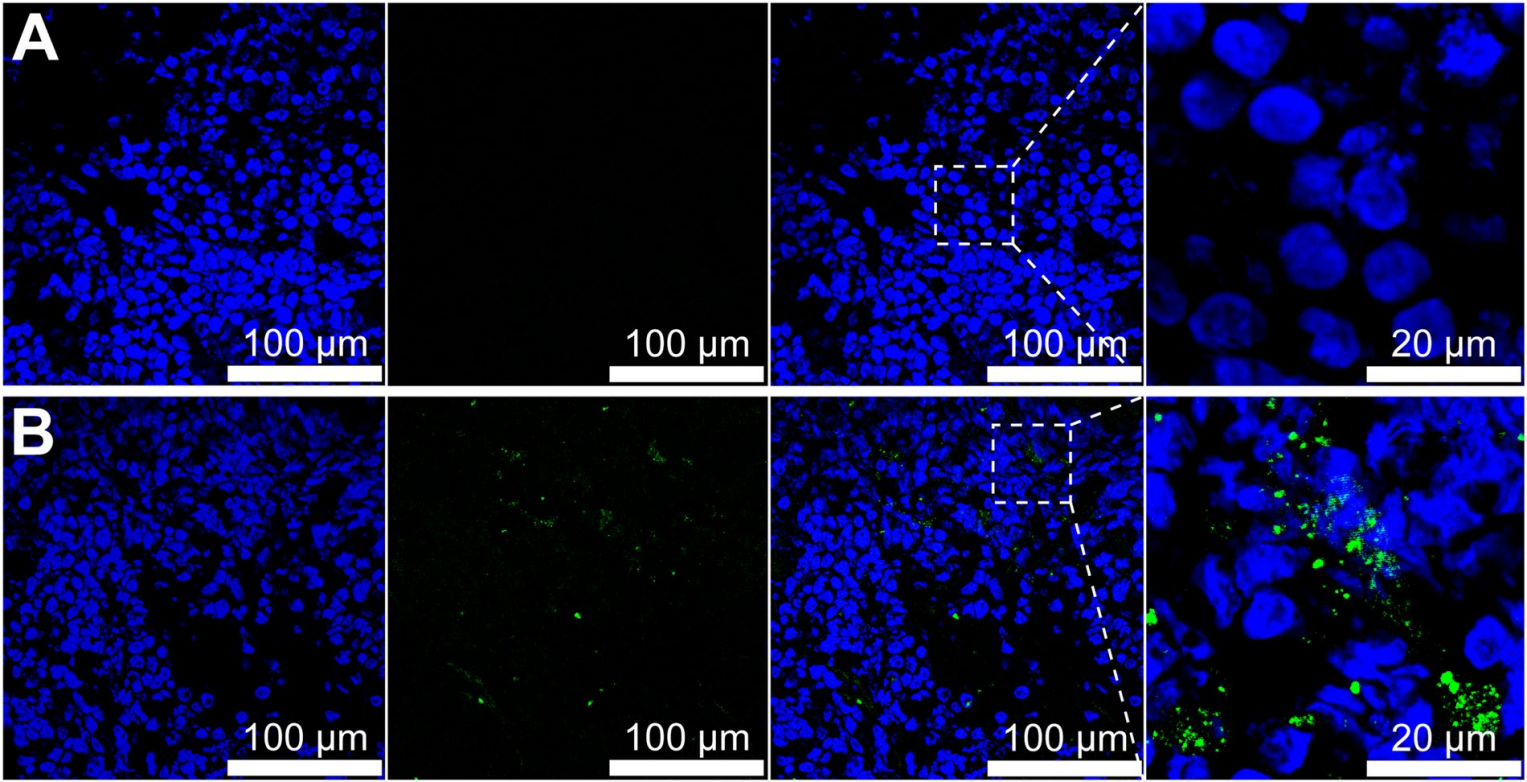
The CLSM images of frozen NCI-H460 tumor tissue of nude mice following injection of Saline (**A**) and PGG-PTX-DTPA-Gd/DiO NPs (**B**). PGG-PTX-DTPA-Gd/DiO NPs were accumulated in the tumor tissue.

The tumor accumulation of PGG-PTX-DTPA-Gd/DiO NPs was observed by CLSM. The results (Fig. 7B) showed that PGG-PTX-DTPA-Gd/DiO NPs could be accumulated in tumor tissue (green fluorescence), which should be due to the EPR effect. The PGG-PTX-DTPA-Gd/DiO NPs showed (Figure S7) almost no distribution in heart and lung, obvious distribution in liver, and a little distribution in spleen and kidney. The distribution in liver and spleen should be caused by the macrophage phagocytosis of NPs, which is a common feature of nano drug delivery systems^34^. The distribution in kidney should be attributed to the elimination of DiO in kidney. The results are almost consistent with that of *in vivo* fluorescent imaging (Fig. 4) and MR imaging (Fig. 5).

## Discussion

In this work, PGG-PTX-DTPA-Gd NPs were prepared by linking Gd-DTPA to PGG-PTX via amide linkages to be used for tumor theranostics. The results showed that the PGG-PTX-DTPA-Gd NPs delivery system could be selectively delivered to the tumor tissue, and showed enhanced relaxivity and significantly inhibited tumor growth. These results indicated that PGG-PTX-DTPA-Gd NPs can be used as a drug delivery system integrated with tumor diagnosis and treatment functions.

For MR imaging, the relaxivity is an important parameter, which determines the contrast ability of a paramagnetic compound, as higher relaxivity (r_1_) contributes to higher signal enhancement. In recent years, with the in-depth research on MRI, contrast agent with high relaxivity has attracted more and more attention. To enhance the contrast between normal tissue and diseased tissue, contrast agent (T_1_ and T_2_) are widely used clinically^35–37^. The T_1_ contrast agent is primarily based on Gd^3+^ and provides a brighter signal, while the T_2_ contrast agent produces a darker signal and is typically Iron Oxide Nanoparticle (IONP)^38–40^. However, the resolution of contrast enhancement imaging on the market are very limited, which should be attributed to the low relaxivity of conventional MRI contrast agents caused by their low molecular weight. Our *in vitro* MR results showed that the relaxivity (r_1_ = 18.98 mM^−1^S^−1^, Fig. 2B) of PGG-PTX-DTPA-Gd NPs are 4.9 times greater than the current clinically available T_1_ contrast agent Gd-DTPA. Furthermore, the *in vivo* MR imaging results showed that the tumor signal ratio of PGG-PTX-DTPA-Gd NPs was 144%, and that of Gd-DTPA was 108%, which further confirmed the increased relaxivity of PGG-PTX-DTPA-Gd NPs (Fig. 5C). The high relaxivity of PGG-PTX-DTPA-Gd NPs may result from both the additive effect of all Gd^3+^ paramagnetic centers in the confined space of PGG-PTX NPs and the reduction of molecular tumbling rates^33, 41^. This result is also consistent with a number of previous studies^42–44^.

In addition, the *in vivo* pharmacokinetic behavior of contrast agents also plays an important role^45^. This mainly included the circulating time and tissue distribution of contrast agents. First, most of the contrast agents on the market have a very short circulating time, which is reasonable as the contrast agent can be eliminated outside in time to prevent accumulation in the body. However, if they are used for tumor theranostics, the short circulating time is often not so reasonable since they cannot even enter tumor tissue and are eliminated outside. Second, if contrast agents are used for tumor diagnosis and treatment, adequate distribution in the tumor tissue is necessary. PGG-PTX-DTPA-Gd NPs we prepared have an increased molecular weight compared with Gd-DTPA, making it difficult to be excreted out and increased the circulating time. Meanwhile, PGG-PTX-DTPA-Gd NPs can be passively targeted into tumor tissue by the EPR effect, which should be a prerequisite for tumor diagnosis and treatment. The above *in vivo* MR imaging results showed a significant enhancement of T_1_-weighted signal in the tumor site of PGG-PTX-DTPA-Gd NPs group at 4 h post-injection (Fig. 5B, the red arrow). Such an enhancement in T_1_-weighted signal could show the location of the tumor and also the distribution of nanoparticles.

In summary, we have successfully developed a PGG-PTX-DTPA-Gd NPs delivery system for tumor theranostics. The multifunctional nano delivery system could inhibit tumor growth via the EPR effect and MRI guided imaging. PGG-PTX-DTPA-Gd NPs delivery system we prepared showed good chemotherapeutic efficacy and tumor diagnostic ability. It may be traced *in vivo* in real time, and could provide timely feedback on the clinical effectiveness of the drug. PGG-PTX-DTPA-Gd NPs are an effective delivery system for tumor theranostics, and should have a potential value in the personalized treatment of tumor.

## Experimental section

### Materials

PGG-PTX nanoconjugate (Poly-(L-γ-glutamyl-glutamine)-paclitaxel) (PTX, 34.9 wt%) were synthesized by our laboratory^26^. *p*-NH_2_-Bn-DTPA (S-2-(4-Aminobenzyl)-diethylenetriamine pentaacetic acid) was purchased from Macrocyclicas (USA). GdCl_3_·6H_2_O, N-hydroxysuccinimide (NHS) and N,N′-dimethylaminopyridine were supplied from Sigma-Aldrich, Inc. Magnevist (Gd-DTPA) was obtained from Bayer Schering Pharma AG (Berlin, Germany). N-(3-dimethylaminopropyl)-N′-ethylcarbodiimide (EDC) was purchased from EMD Chemicals Inc. (Darmstadt, Germany). All other chemicals and reagents were commercially available and directly used.

Human NCI-H460 carcinoma cell line was obtained from ATCC. Cell Counting Kit-8(CCK-8) was purchased from DOJINDO Laboratorise chemical technology (Shanghai) co., LTD. DiO (3,3′-dioctadecyloxacarbocyanine, perchlorate) and DiR (1,1′-dioctadecyl-3,3,3′,3′-tetramethyl indotricarbocyanine Iodide) were purchased from Tianjin Biolite Biotech Co., LTD. Hoechst 33342 was purchased from Beyotime Institute of Biotechnology.

### Animals

All experiments involving animals were performed in accordance with the guidelines of the Institutional Animal Care and Use Committee (IACUC) of East China Normal University. All experimental protocols were approved by the IACUC of East China Normal University. Male Balb/c nude mice (four weeks old) were obtained from SLAC Ltd (Shanghai, China) and maintained under SPF conditions. All efforts were made to minimize the number of animals used and their suffering. Animal experiments were reported in accordance with the ARRIVE (Animal Research: Reporting *In Vivo* Experiments) guidelines.

Building of tumor animal models. Male nude mice (6 weeks) were inoculated subcutaneously with NCI-H460 cells (5 × 10^6^ cells in 0.1 mL PBS). Tumor diameters were measured every two days using a sliding caliper. The tumor volume (TV) was calculated according to the formula TV = (L × W^2^)/2, where L and W were the length of the major and minor diameters, respectively.

Evaluation of antitumor effect. The tumor-bearing mice were randomly divided into three groups (n = 6): 1) saline; 2) PGG-PTX NPs (20 mg/kg of PTX); 3) PGG-PTX-DTPA-Gd NPs (20 mg/kg of PTX). The drug administration was started when the tumors reached an average volume of 100–120 mm^3^ ^46^. The three groups of mice were administered intravenously via tail-vein. The body weight and tumor volume of the mice were measured every two days for until the TV of the saline group reached 3000 mm^3^, when all the mice were terminated.

*In vivo* fluorescence imaging. The accumulation of PGG-PTX-DTPA-Gd NPs in tumor tissue was evaluated by near-infrared *in vivo* imaging study. The tumor-bearing mice (n = 3) were i.v. injected with 100 µL of PGG-PTX-DTPA-Gd/DiR via the tail vein. At different time points of pre-injection, 1, 2, 4, 8, 12, 24, 48 h post-injection, the mice were anesthetized, and the fluorescent images were captured using an *in vivo* imaging system (*In-Vivo* FX PRO, Bruker) equipped with a DiR filter sets (excitation/emission, 730/790 nm). The tumor tissues were harvested and weighed at the end of the experiment. Pharmacokinetic profile of PGG-PTX-DTPA-Gd/DiR NPs in NCI-H460-bearing nude mice was obtained based on the semi-quantitative ROI analysis of the fluorescent images.

*In vivo* MR imaging. The mice (n = 3) were anesthetized by intraperitoneal injection of pentobarbital sodium (50 μL, 2.5%, 50 mg/kg). MR images were acquired on a 3 T MRI (SIEMENS MAGNETOM Trio I-class, Germany) scanner with an animal coil using a 2D T_1_-weighted spin-echo sequence. PGG-PTX-DTPA-Gd NPs were injected at a dose of 0.07 mmol-Gd/kg body weight via the tail vein, and Gd-DTPA was as the control with the same dose. Scans were performed at different time points. The sequence parameters used for the image acquisition were as follows: TR = 500 ms, TE = 14 ms, α = 90°, FOV = 50 (100, 50%) mm, slice thickness = 1 mm, and image matrix = 384 × 384. The MRI T_1_ signal intensities (SI) within the regions of interest (ROIs) were measured three times before and after injection of the PGG-PTX-DTPA-Gd NPs. The relative enhancement signal intensity (RESI) was calculated according to the following formula: RESI (%) = SI_contrast_/SI_pre_ × 100%, in which, SI_contrast_ is the signal intensity of tumor after the injection, and SI_pre_ is that before the injection.

### Synthesis of PGG-PTX-DTPA-Gd

PGG-PTX-DTPA-Gd conjugate was synthesized as shown in Figure S1. PGG-PTX-DTPA-Gd were prepared by chemical synthesis in two steps starting from PGG-PTX. Two intermediate compounds were isolated and characterized before used. PGG-PTX-DTPA-Gd were obtained by dialysis with a tangential flow filtration system followed by lyophilization.

### Synthesis of PGG-PTX-NHS

PGG-PTX sodium salt (1.0 g) was dissolved in deionized water (50 mL) and acidified with hydrochloric acid solution (0.2 M). The solution was desalted by dialysis with a membrane (10 k Da cutoff) against deionized water. PGG-PTX, a colorless solid, was recovered after evaporating water under vacuum.

750 mg of PGG-PTX was stirred in 50 mL of anhy DMF for half an hour. Then N-hydroxysuccinimide (NHS, 750 mg) and N-(3-dimethylaminopropyl)-N′-ethylcarbodiimide (EDC, 750 mg) were added and stirred for 24 h. The reaction mixture was poured into anhydrous ethanol to quench the reaction. The formed precipitate was collected by centrifuge, washed with cold ethanol three times, and dried under vacuum. Then 0.72 g of PGG-PTX-NHS was obtained (yield: 96%).

### Synthesis of PGG-PTX-DTPA

300 mg of activated PGG-PTX-NHS was stirred in anhy DMF (20 mL) for 10 min to give clear solution. Then 30 mg *p*-NH_2_-Bn-DTPA and 78 mg DMAP was added and stirred for 24 h. The reaction was quenched by being poured into 0.2 M HCl solution (50 mL). The formed precipitate was collected by centrifuge and then dissolved with 0.3 M NaHCO_3_ solution (50 mL). The solution was stirred for 15 min and dialyzed (M.W. CO 10 K) for 24 h against water, filtered and lyophilized to obtain PGG-PTX-DTPA (230 mg, 77% yield).

### Synthesis of PGG-PTX-DTPA-Gd

Into 10 mL of sodium acetate-buffered aqueous solution (0.1 M pH 5.5) of PGG-PTX-DTPA (180 mg) was added 80 mg (Gadolinium (III) chloride hexahydrate) in 2 mL 0.1 M sodium acetate solution. The mixed solution was stirred overnight. Xylenol orange indicator was added into the solution, and EDTA (0.1 M) was then added dropwise until the pink color disappeared. The resulting solution was subject to a G50 gel column using an AKTA purifier (GE, USA) and PGG-PTX-DTPA-Gd was collected and lyophilized to yield 160 mg sponge-like powder (yield: 89%).

### Preparation and characterization of PGG-PTX-DTPA-Gd NPs

PGG-PTX NPs and PGG-PTX-DTPA-Gd NPs were prepared by self-assembly method in the solution^26^. The ^1^H-NMR spectra of PGG-PTX and PGG-PTX-DTPA-Gd in deuterated water (D_2_O) were recorded with a Bruker spectrometer at 400 MHz. The gadolinium content of the PGG-PTX-DTPA-Gd was determined by inductively coupled plasma optical emission spectroscopy at 342.2 nm (ICP-OES, IRIS Intrepid II XSP, Thermo Fisher Scientific, USA). The GPC spectra and Mw of PGG-PTX and PGG-PTX-DTPA-Gd were characterized by gel permeation chromatography with a GPC-MALS system (Wyatt, Santa Barbara, California).

### Particle size measurement

The particle size of PGG-PTX NPs and PGG-PTX-DTPA-Gd NPs were determined by dynamic light scattering (DLS) method using a Mastersizer2000 (Malvern Instruments Inc, UK) equipped with He-Ne laser (4 mW, 633 nm) light source and 90° angle scattered-light collection configuration. The NPs (10 mg) were suspended in 1 × PBS (5 mL) and measured for 10 minutes in triplicate at room temperature.

### Morphology of PGG-PTX NPs and PGG-PTX-DTPA-Gd NPs

The morphology of PGG-PTX NPs and PGG-PTX-DTPA-Gd NPs were observed using a transmission electron microscope (TEM) (JEM-2100, Hitachi, Tokyo, Japan) at an accelerating voltage of 75 kV. Negative staining was performed as described previously^47^.

### Characterization of r_1_ relaxivity

The proton longitudinal relaxivity r_1_, which is a parameter to evaluate the ability of contrast agents for MRI^48^. The T_1_ relaxation times of probes at different Gd concentrations were measured with a 3 T MRI (SIEMENS MAGNETOM Trio I-class, Germany). The corresponding r_1_ was calculated from the slope of the linear curve of inverse relaxation time (1/T_1_) as a function of the Gd concentration. The PGG-PTX-DTPA-Gd NPs was diluted with distilled water at Gd concentration range from 0.1 to 0.5 mM, and free Gd-DTPA was as the control at the same dose. The samples were transferred to a 96-well plate, and T_1_ relaxation time was measured with the following parameters: TR = 7000 ms, TE = 11 ms, TI = 24, 100, 200, 400, 600, 900, 1200, 2000, 3000 and 5000 ms, FOV = 120 × 85 mm, average = 1.

### *In vitro* cellular uptake

#### Preparations of PGG-PTX/DiO NPs and PGG-PTX-DTPA-Gd/DiO NPs

PGG-PTX/DiO NPs were prepared by the emulsification-solvent evaporation method^49^. Briefly, DiO was suspended in the mixture of methylene chloride and acetone (3:1, v/v) as the, and PGG-PTX NPs were suspended in sodium cholate solution as the water phase. The organic phase and water phase was mixed and emulsified by ultrasonic method in ice bath. The organic solvent was removed by rotary evaporation to obtain the PGG-PTX/DiO NPs, which were then purified to remove the free DiO by G50 gel column with AKTA purifier.

### Cellular uptake

Cellular uptake study was performed with NCI-H460 cell line. Cells were seeded in glass bottom dish or 6-well plates at a density of 3 × 10^4^ cells per well and cultured for 24 h. Then cells were washed with phosphate buffered saline (PBS) and treated with different concentrations of PGG-PTX/DiO and PGG-PTX-DTPA-Gd/DiO NPs for 4 h.

To observe the cellular uptake of NPs qualitatively, the treated cells were washed three times with cold PBS, then fixed with 4% paraformaldehyde, stained with Hoechst33342 and observed using a confocal laser scanning microscope (CLSM, TCS SP5, Leica, Germany).

For quantitative analysis, the treated cells were washed with cold PBS, trypsinized and harvested by centrifugation at 1200 rpm for 5 min. The cells were resuspended in 200 μL PBS and filtered through a 40 mm nylon mesh to remove cell aggregates. The cell suspension was then analyzed by flow cytometry (Guava easyCyte, USA).

### *In vitro* PTX release

To investigate the release profile of PTX from PGG-PTX NPs and PGG-PTX-DTPA-Gd NPs, we used sodium salicylate solution (0.8 M, pH 6.5) as the dissolution medium as reported previously^50^. A series of 20 mL vials containing exactly 1.0 mL of each working solution were prepared. The samples of PGG-PTX NPs and PGG-PTX-DTPA-Gd NPs were added to reach a final concentration of 2 mg/mL. Then these vials were shaken horizontally at 120 min^−1^ with an incubator shaker at 37 °C. The sample was withdrawn at predetermined time points and mixed with 2 mL ethyl acetate to extract PTX. The ethyl acetate solution was dried with nitrogen blowing instrument. Then the samples were resuspended with acetonitrile and filtered through a 0.22 μm pore-sized filtration membrane. The injection volume was 20 μL and the flow rate of mobile phase was 1.0 mL/min. Analysis of the drug release was performed using an Agilent 1100 series HPLC system (Agilent Technologies, Santa Clara, CA, USA).

### *In vitro* cytotoxicity assays

The *in vitro* cytotoxicity of NPs was investigated by the CCK-8 assay according to the published protocols with modifications^51^. NCI-H460 cells (1.0 × 10^4^) were seeded in 96-well plates and incubated for 24 h. Then serial dilutions of PGG-PTX NPs and PGG-PTX-DTPA-Gd NPs were added to the plate (100 μL/well). Following incubation for up to 48 hours, the cells were treated with 10 μL of CCK-8 solution and cultured for 4 h. The absorbance was measured with a microplate reader (SpectraMax M5, Molecular Devices, USA) at 450 nm. The survival rate was calculated using the following formula: viability rate (%) = (OD_test group_ − OD_Blank_)/(OD_control group_ − OD_Blank_) × 100%.

### Histological verification

#### H&E staining

The tumor bearing mice after the treatment with Saline (control), PGG-PTX NPs and PGG-PTX-DTPA-Gd NPs at the equivalent PTX dose of 20 mg/kg. The body weight of mice was recorded every 2 days and during four-week feeding. Then, the mice were sacrificed and the histocompatibility was evaluated by Hematoxylin and Eosin (H&E) staining^52^. Tumor tissues and main organs were collected and immediately fixed using 10% formalin solution and paraffin embedded tissues. Routine paraffin sections and H&E staining were performed according to standard clinical pathology protocols.

#### TUNEL assay

Tissue apoptotic cells were detected with TUNEL (TdT-mediated dUTP Nick-End Labeling) using a commercial kit (No. 12156792910; Roche, Switzerland) according to the manufacturer’s protocol. TUNEL assay was performed for paraffin sections fixed with 4% paraformaldehyde and processed^53^. The sections were analyzed under a fluorescent microscope (Olympus, IX71, Japan).

### Tumor accumulation

The PGG-PTX-DTPA-Gd/DiO NPs were i.v. injected to tumor-bearing nude mice. The mice (n = 3) were sacrificed 4 h post-injection, and the tumor tissues and main organs (including heart, liver, spleen, lung, and kidney) were collected, fixed, dehydrated, and frozen in acetone/dry ice mixture. The frozen samples were further cut to sections (10 μm) with a cryostat (CM3050 S, Leica, Germany). The sections were stained with Hochest33342 and washed with PBS, and then observed using a confocal laser scanning microscope (CLSM, TCS SP5, Leica, Germany).

### Statistical analysis

Statistical differences were evaluated with two-tailed student’s t-test and one-way ANOVA. The differences were considered to be significant at P < 0.05 and very significant at P < 0.01.

## Electronic supplementary material

Supporting Information Revised
