# Supplementary material for: A Novel Gd-DTPA-conjugated Poly(L-γ-glutamyl-glutamine)-paclitaxel Polymeric Delivery System for Tumor Theranostics
Source: Sci Rep. 2017 Jun 19;7:3799. doi: 10.1038/s41598-017-03633-9 (PMC5476566; doi:10.1038/s41598-017-03633-9)
Supplement: Supplementary file 1 — Supporting Information Revised [file 41598_2017_3633_MOESM1_ESM.pdf]

Supporting Information

A Novel Gd-DTPA-conjugated Poly(L-γ-glutamyl-glutamine)-paclitaxel Polymeric Delivery System for Tumor Theranostics

Lipeng Gao[1], Jinge Zhou[1], Jing Yu[1], Qilong Li[1], Xueying Liu[1], Lei Sun[1], Ting Peng[1], Jing Wang[1], Jianzhong Zhu[1], Jihong Sun[2], Weiyue Lu[3], Lei Yu[1], Zhiqiang Yan[1] & Yiting Wang[1]

[1]Institute of Biomedical Engineering and Technology, Shanghai Engineering Research Center of Molecular Therapeutics and New Drug Development, School of Chemistry and Molecular Engineering, East China Normal University, Shanghai 200062, China.
[2]Department of Radiology, Sir Run Run Shaw Hospital, School of Medicine, Zhejiang University, Hangzhou 310016, China.
[3]Department of Pharmaceutics, School of Pharmacy, Fudan University & Key Laboratory of Smart Drug Delivery, Fudan University, Ministry of Education, Shanghai 201203, China.

Correspondence and requests for materials should be addressed to Z.Y. (email: zqyan@sat.ecnu.edu.cn), Y.W. (email: ytwang@nbic.ecnu.edu.cn) or J.S. (email: braversun@sina.com)

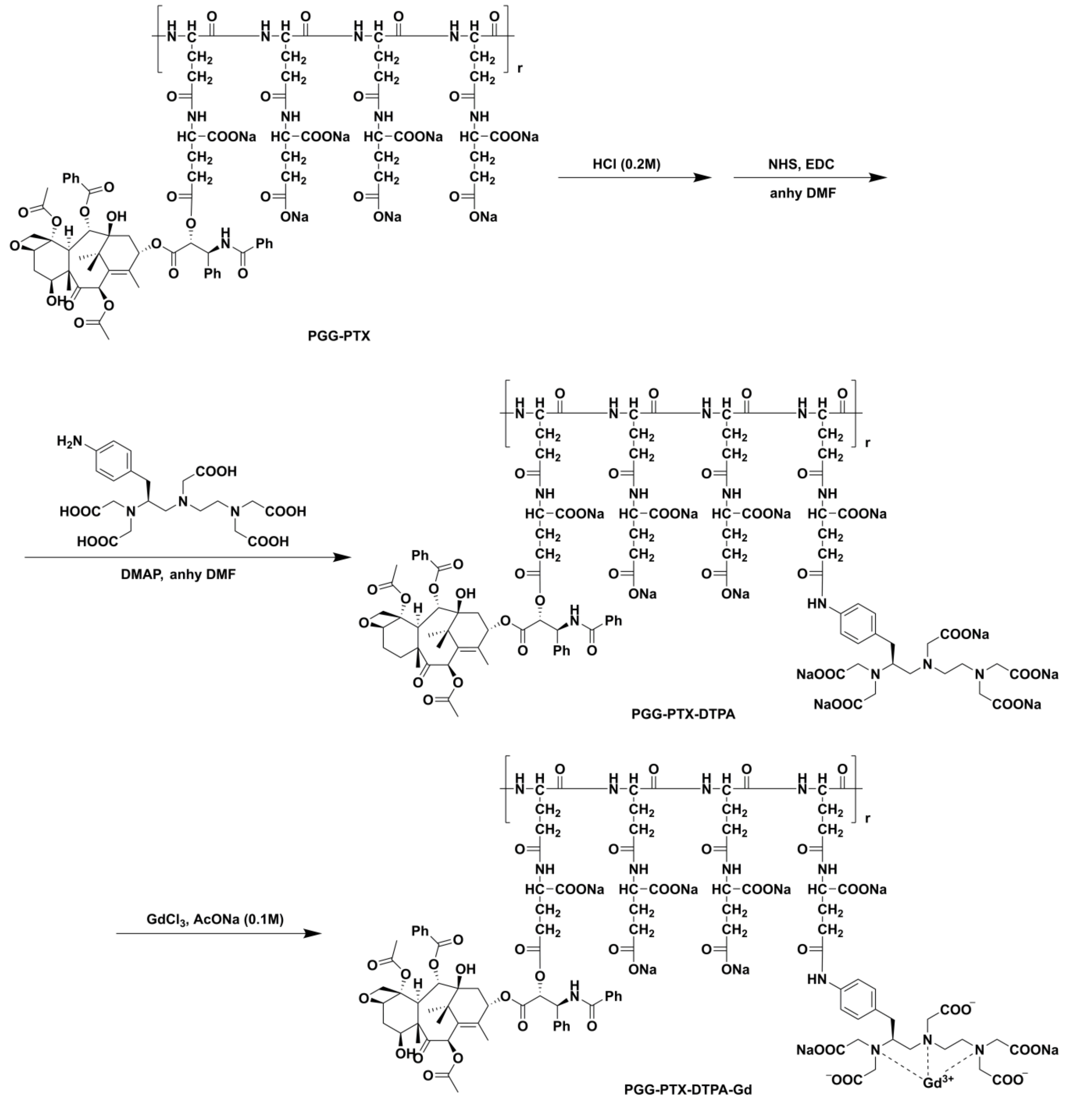

Figure S1. PGG-PTX-DTPA-Gd synthetic scheme.

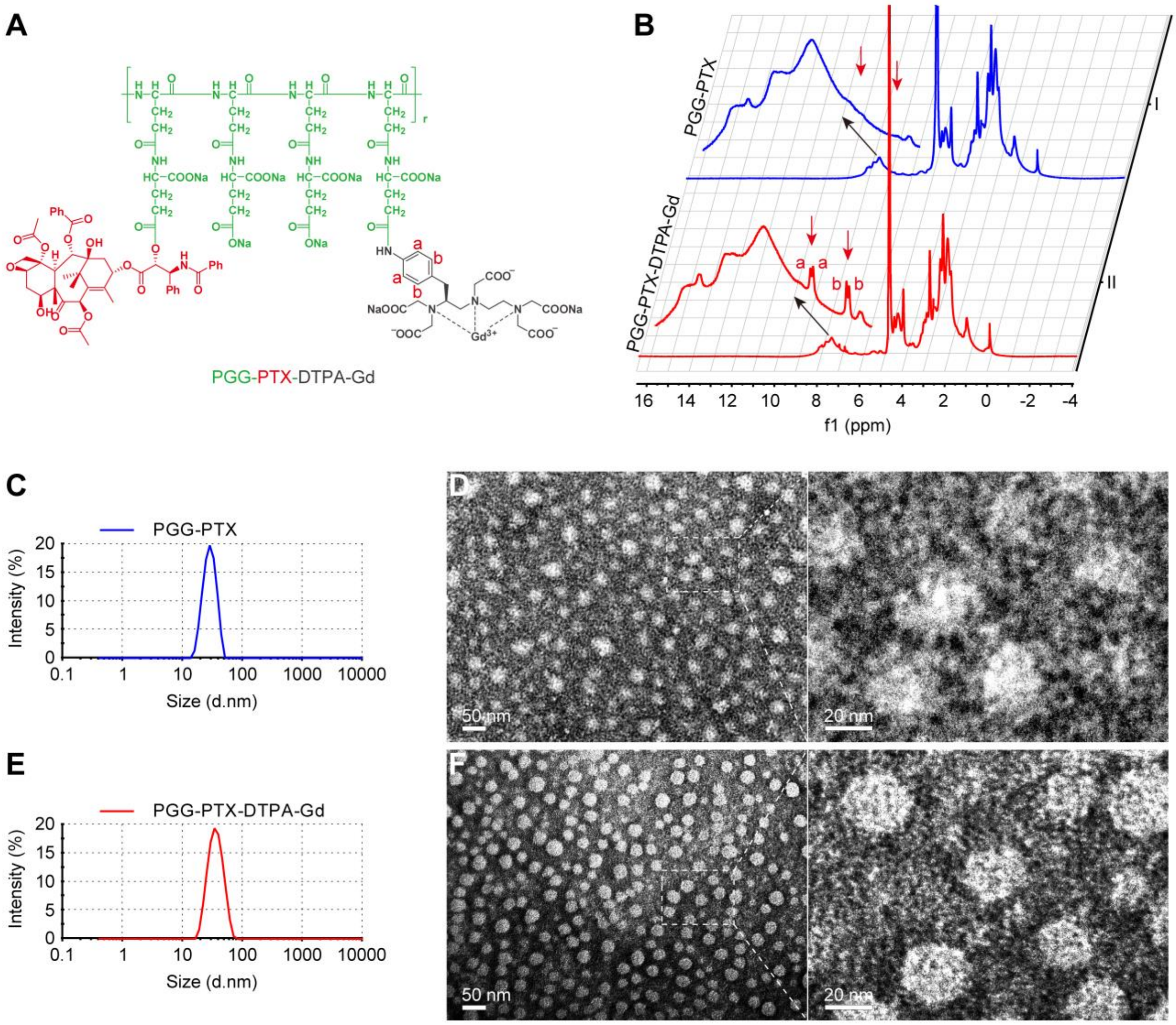

Figure S2. Characterization of the PGG-PTX-DTPA-Gd nanoparticles. (A) The chemical structure of the PGG-PTX-DTPA-Gd conjugate. The green part is PGG, the red is PTX, and the black is Gd-DTPA. (B) ^{1}H-NMR spectra of PGG-PTX NPs (I) and PGG-PTX-DTPA-Gd NPs (II). The characteristic peaks of *p*-NH_2-Bn-DTPA at 7.07 (d, J = 7.8 Hz, 2H) and 6.81 (d, J = 7.8 Hz, 2H) ppm (a and b, the red arrows) showed that *p*-NH_2-Bn-DTPA and PGG-PTX NPs were successfully linked together. The particle size and polydispersity of the PGG-PTX NPs and PGG-PTX-DTPA-Gd NPs obtained from DLS (C, E), and TEM (D, F). The Z-mean diameter of PGG-PTX NPs and PGG-PTX-DTPA-Gd NPs were approximately 29.2 nm and 35.9 nm and exhibited uniform spherical morphology as displayed in the TEM images.

Table S1

Characterization of polymer conjugates.

Sample	Mw (kDa)	Mw/Mn (PDI)
PGG-PTX	79.17	1.25
PGG-PTX-DTPA-Gd	93.89	1.38

All data are expressed as the mean Mw of the samples (n = 5).

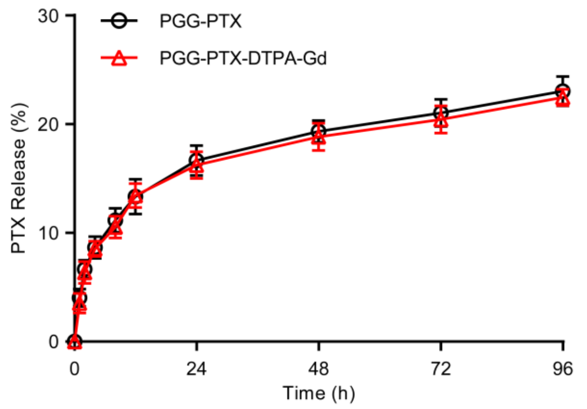

Figure S3. Release of PTX from PGG-PTX NPs and PGG-PTX-DTPA-Gd NPs as function of time in sodium salicylate solution at 37 ℃ (n = 3, bars represent means ± SD). There was no significant difference in PTX release between PGG-PTX NPs and PGG-PTX-DTPA-Gd NPs on all time points.

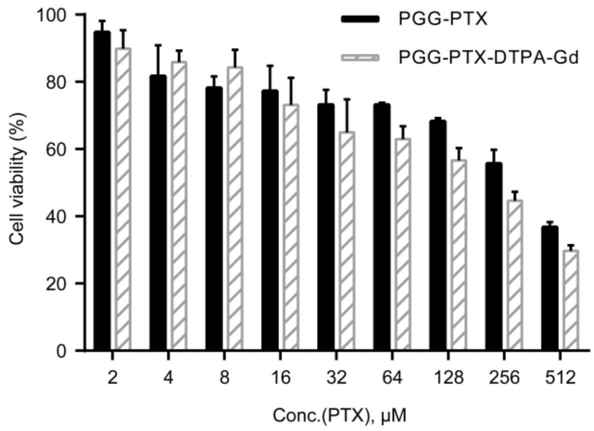

Figure S4. The cytotoxicity of PGG-PTX NPs and PGG-PTX-DTPA-Gd NPs on NCI-H460 cells as measured by CCK-8 assay. PGG-PTX-DTPA-Gd NPs showed similar cellular cytotoxicity with PGG-PTX NPs.

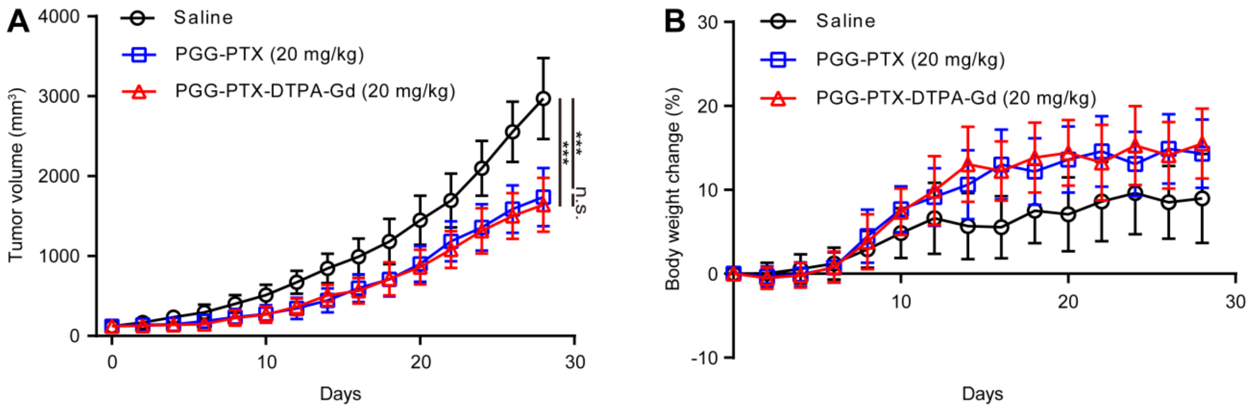

Figure S5. Antitumor efficacy in nude mice bearing NCI-H460 human non-small cell lung carcinoma tumors. Mean tumor growth curves (A); Body weight change (B). PGG-PTX NPs and PGG-PTX-DTPA-Gd NPs had effective antitumor effect in vivo.

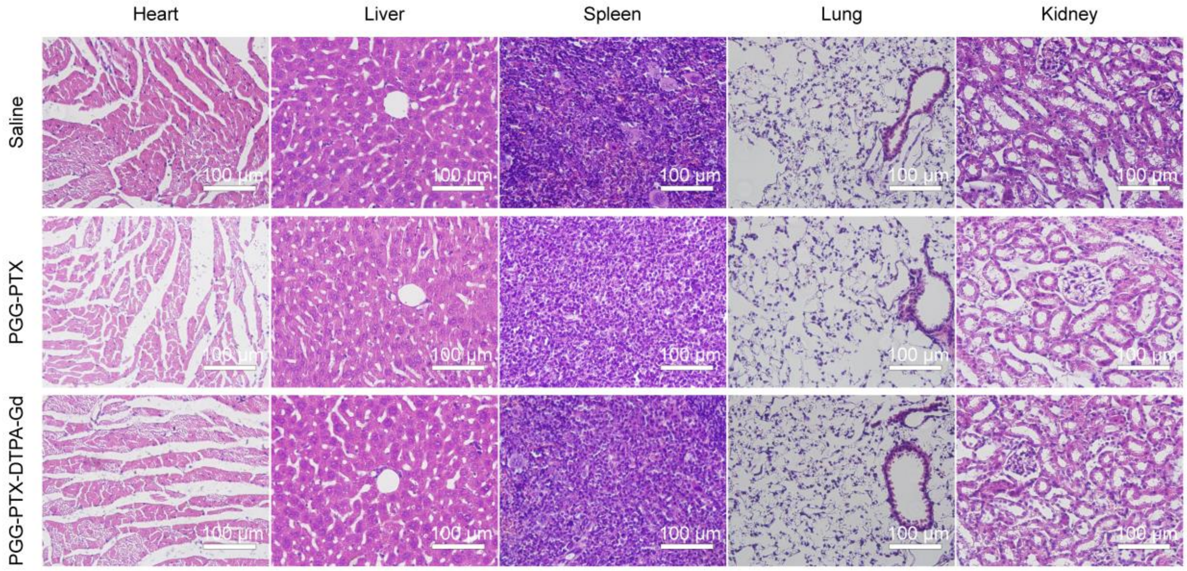

Figure S6. The H&E staining for different organs of tumor bearing mice after the treatment (40×) with Saline (control), PGG-PTX NPs and PGG-PTX-DTPA-Gd NPs. The main organs (including heart, liver, spleen, lung, and kidney) of the two NPs groups showed no obvious pathological abnormity compared with those of Saline groups.

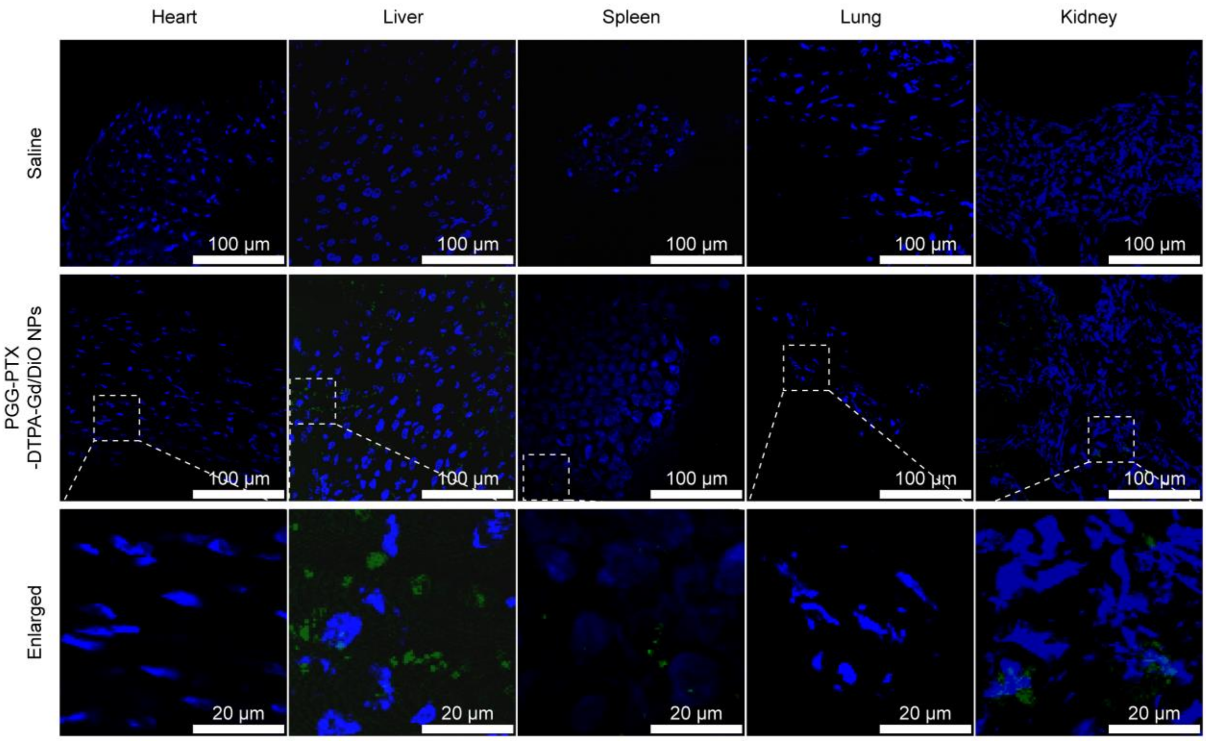

Figure S7. The CLSM images of frozen the main organs of NCI-H460 nude mice following injection of Saline and PGG-PTX-DTPA-Gd/DiO NPs.
